# Atypical Dengue Fever Presentation With Severe Leukopenia: A Case Report From an Endemic Border Region

**DOI:** 10.7759/cureus.91359

**Published:** 2025-08-31

**Authors:** Elias Arellano Villanueva, Alhasan Asaad, Jose E Campo Maldonado

**Affiliations:** 1 Department of Internal Medicine, The University of Texas Rio Grande Valley School of Medicine, Edinburg, USA; 2 Department of Internal Medicine, Valley Baptist Medical Center, Harlingen, USA; 3 Department of Infectious Disease, The University of Texas Rio Grande Valley School of Medicine, Edinburg, USA

**Keywords:** atypical presentation, bone marrow biopsy, border health, cytopenias, dengue fever, endemic disease, hematemesis, neutropenia, severe leukopenia, south texas

## Abstract

Dengue virus infection is increasingly recognized in South Texas, particularly in areas bordering Mexico. Although many cases are mild and self-limiting, dengue can present with atypical features, including hematological abnormalities and bleeding, even in the absence of plasma leakage. A 22-year-old Hispanic woman presented to a hospital in South Texas with hematemesis, fever, and gastrointestinal symptoms following travel to northeastern Mexico (Monterrey, Nuevo Leon). Initial laboratory workup revealed severe leukopenia and neutropenia, mild thrombocytopenia, and elevated liver enzymes, but no evidence of plasma leakage. A bone marrow biopsy was performed due to persistent cytopenias, revealing a reactive process consistent with viral infection. The patient improved with supportive care, including hydration, antipyretics, and symptom management, which reflects the mainstay of dengue management. This case illustrates an atypical dengue presentation marked by hematological derangements without classic features, such as hemoconcentration or plasma leakage. It underscores the diagnostic challenge dengue poses in non-typical cases, the importance of maintaining clinical suspicion in endemic areas, and the need for judicious use of invasive investigations and blood product support. Dengue may present atypically with severe cytopenias and bleeding without plasma leakage. Clinicians in endemic areas such as South Texas should consider dengue in febrile patients with cytopenias, even in the absence of plasma leakage, and avoid unnecessary interventions when supportive care suffices.

## Introduction

Dengue virus infection is the most common arthropod-borne viral disease globally, caused by one of four single-stranded RNA viruses (DENV 1-4) of the *Flavivirus *genus and transmitted primarily by *Aedes aegypti* and *Aedes albopictus* mosquitoes [1,2]. Dengue fever, also called breakbone fever, typically presents with high fever, joint pain, and muscle spasms [1,2]. While many infections are asymptomatic, clinical dengue can range from mild febrile illness to severe dengue hemorrhagic fever and dengue shock syndrome [3]. In recent decades, dengue has re-emerged in the United States, particularly in the Rio Grande Valley (RGV) of South Texas, which shares ecological and social characteristics with endemic regions of northern Mexico [4,5]. All four DENV serotypes have been detected in the region, and sporadic outbreaks of locally acquired dengue have occurred, with notable variability in seroprevalence between border communities [6,7]. For instance, in 2004, the dengue seroprevalence was 78% in Matamoros, Mexico, just across the border from Brownsville, Texas, where it was only 2% [7,8]. Between 1980 and 1999, over 65,000 dengue cases were reported in northern Mexico, while only 64 cases were documented in the US side during the same period [9,10]. Globally, 2.5 billion people live in endemic areas, and increased mobility has contributed to its spread into previously non-endemic regions [11]. Despite this growing threat, dengue often goes unrecognized in the US due to its nonspecific clinical features and a general lack of clinical suspicion in areas not historically considered endemic [11,12]. Hematological complications are prominent, including thrombocytopenia, leukopenia, and anemia [12]. Severe cases may involve plasma leakage, defined as the movement of fluid from the intravascular space into the surrounding tissues, clinically identified through hemoconcentration, pleural effusion, ascites, or hypoproteinemia and frequently accompanied by bleeding and organ impairment [11]. Lymphohistiocytosis, often underdiagnosed, carries a risk of fatal outcomes [12]. Dysregulated immune responses are central to dengue pathogenesis, with thrombocytopenia and coagulopathy being major causes of bleeding in severe cases [13]. We report a case of dengue in a young adult with an atypical presentation involving severe leukopenia and hematemesis, but without plasma leakage. This case highlights the need for heightened diagnostic awareness and public health preparedness in South Texas.

This work was previously presented as a poster at the 3rd International Conference on Cancer Health Disparities (ICCHD) and the UTRGV School of Medicine Research Symposium 2025, held on February 14-15, 2025, in Mission, Texas.

## Case presentation

A 22-year-old Hispanic female with no significant past medical history presented to the emergency department (ED) in South Texas with hematemesis. Two days prior to presentation, while visiting family in Monterrey, Mexico, she developed a high fever (maximum temperature: 107.6°F or 42°C), chills, diarrhea, vomiting, and severe headache with photophobia. She sought care at a private clinic in Matamoros, Mexico, where she tested positive for the dengue virus via NS1 antigen assay. She received symptomatic treatment, including fluids, acetaminophen, and multivitamins, but her symptoms persisted.

During her return trip to San Benito, Texas, she experienced an episode of bright red hematemesis, prompting immediate presentation to a local hospital. Initial examination revealed petechiae on the right arm and ecchymosis over the right upper gluteal area. Laboratory findings were significant for sepsis secondary to dengue fever, severe leukopenia (white blood cell count nadir of 0.7 × 10⁹/L), neutropenia, mild thrombocytopenia, transaminitis (ALT and AST >500 U/L and >1,000 U/L, respectively), and microscopic hematuria, as shown in Table 1. Additional diagnostic testing results are summarized in Table 2.

**Table 1:** Hematological findings of the patient during the hospital stay The table shows progressive leukopenia, neutropenia, and thrombocytopenia, with the WBC count reaching a nadir on day three and platelet counts declining to a low of 58 × 10⁹/L by day five. Both parameters gradually recovered by discharge, paralleling clinical improvement. Peak elevations in AST and ALT occurred on hospital days 2–5, all of which resolved with supportive care. MCV: mean corpuscular volume; MCH: mean corpuscular hemoglobin; MCHC: mean corpuscular hemoglobin concentration; HCT: hematocrit; ALP: alkaline phosphatase; ALT: alanine transaminase; AST: aspartate aminotransferase; BUN: blood urea nitrogen

Laboratory Studies	Reference Range	Day 1	Day 2	Day 3	Day 4	Day 5	Day 6	Day 7
Erythrocytes (*10^12^/L)	4.2–5.9	1.50	0.90	0.70	0.80	1.70	2.20	2.70
Hemoglobin (g/L)	120–160	11.60	11.10	11.10	12.80	11.30	10.80	10.30
Hematocrit (%)	36–50	35.00	33.40	32.50	37.70	33.10	32.00	31.20
MCV (fL)	80–100	86.60	85.60	84.00	84.00	84.00	84.00	85.20
MCH (pg)	27–33	28.70	28.50	28.70	28.50	28.70	28.30	28.10
MCHC (g/L)	320–360	33.10	33.20	34.20	34.00	34.10	33.80	33.00
Platelet Count (*10^9^/L)	150–400	141.00	104.00	85.00	70.00	58.00	78.00	132.00
Neutrophil (%)	40–70	82.80	-	45.80	35.10	10.40	10.00	10.60
Lymphocyte (%)	20–40	11.00	-	45.70	51.90	78.50	78.90	74.90
Monocyte (%)	2–8	4.80	-	7.10	10.40	9.90	10.10	13.70
Eosinophil (%)	1–4	0.00	-	0.00	0.00	0.00	0.00	0.00
Basophil (%)	0–1	0.70	-	0.00	1.30	0.60	0.50	0.40
Glucose (mmol/L)	3.9–5.6	98.00	86.00	93.00	100.00	98.00	107.00	114.00
BUN (mmol/L)	2.5–7.1	5.00	4.00	4.00	4.00	4.00	5.00	9.00
Creatinine (µmol/L)	44–106	0.49	0.49	0.45	0.41	0.33	0.41	0.46
Albumin (g/L)	35–50	4.10	3.70	3.50	3.10	3.00	3.00	3.10
Total bilirubin (µmol/L)	5–21	0.90	0.90	0.80	1.10	0.80	0.80	0.80
Conjugated Bilirubin	0–7	-	-	-	1.10	0.80	0.50	0.40
ALP (U/L)	44–147	122.00	113.00	110.00	123.00	134.00	151.00	149.00
ALT (U/L)	7–56	202.00	>500	>500	>500	>500	>500	483.00
AST (U/L)	10–40	245.00	>1000	>1000	>1000	>1000	810.00	490.00
Protein (g/L)	6.0–8.3	6.60	6.10	5.70	5.10	5.00	5.20	5.30

**Table 2:** Hepatitis virology and autoimmune workup Summary of the patient’s negative autoimmune and infectious workup, supporting dengue as the primary etiology of her cytopenias. IgM: immunoglobulin M; IgG: immunoglobulin G; HBsAg: hepatitis B surface antigen; ANA: antinuclear antibodies; Ab: antibody, EBV: Epstein-Barr virus; Fvr: fever

Laboratory Investigations	Results	
ANA	Negative	
CMV DNA Quant	Negative	
Dengue Fvr IgG	1.28	
Dengue Fvr IgM	1.12	
EBV Early IgG	<9.0	
EBV Ab VCA IgM	<36.0	
Influenza A Ag	Negative	
Influenza B Ag	Negative	
HepA IgM	Nonreactive	
HepB Core IgM	Nonreactive	
HBsAg	Nonreactive	
Hep C Ab	Nonreactive	
HIV 1/2 Ab Screen	Negative	
A-1 Antytripsin DNA	Negative	
Mitochondrial Ab	Negative	
Mononucleosis Screen	Negative	
Smooth Muscle Ab	Negative	
Typhus Fever IgG	Negative	

She was admitted for further evaluation and management. An infectious disease (ID) consult was obtained, and empiric doxycycline (100 mg PO Q12H) was initiated due to concerns for Rickettsial infections. She received intravenous normal saline 0.9% (1,000 mL over 10 hours). Supportive care included acetaminophen (650 mg PO Q4H PRN for fever), ondansetron (4 mg IV push Q8H PRN for nausea), Dilaudid (0.5 mg IV push Q4H PRN for severe pain), pantoprazole (40 mg IV push daily), and potassium chloride (40 mEq PO) for electrolyte replenishment. Neutropenic precautions were implemented on hospital day three.

Initial complete blood count (CBC) from the Mexican clinic showed hemoglobin of 12.4 g/dL, hematocrit of 39%, and platelet count of 228 × 10⁹/L. Repeat CBCs during hospitalization revealed progressive leukopenia, neutropenia, and thrombocytopenia (Table 1). A peripheral blood smear demonstrated normocytic normochromic red blood cells, teardrop cells, and large platelets. Given the persistent cytopenias, hematology/oncology consultation recommended a bone marrow biopsy.

**Figure 1:**
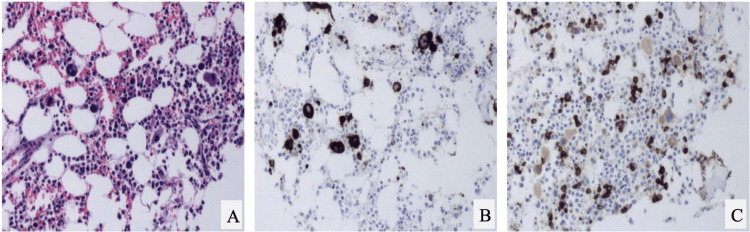
Bone marrow biopsy revealing reactive marrow changes in dengue-associated cytopenias (A) H&E stain at medium power shows a mildly hypocellular marrow with preserved myeloid elements and decreased erythroid precursors. (B) CD42b immunostain highlights increased megakaryocytes, consistent with megakaryocytic hyperplasia. (C) CD71 immunostain demonstrates reduced erythroid lineage cells, supporting erythroid hypoplasia. H&E: hematoxylin and eosin; CD: cluster of differentiation

**Figure 2:**
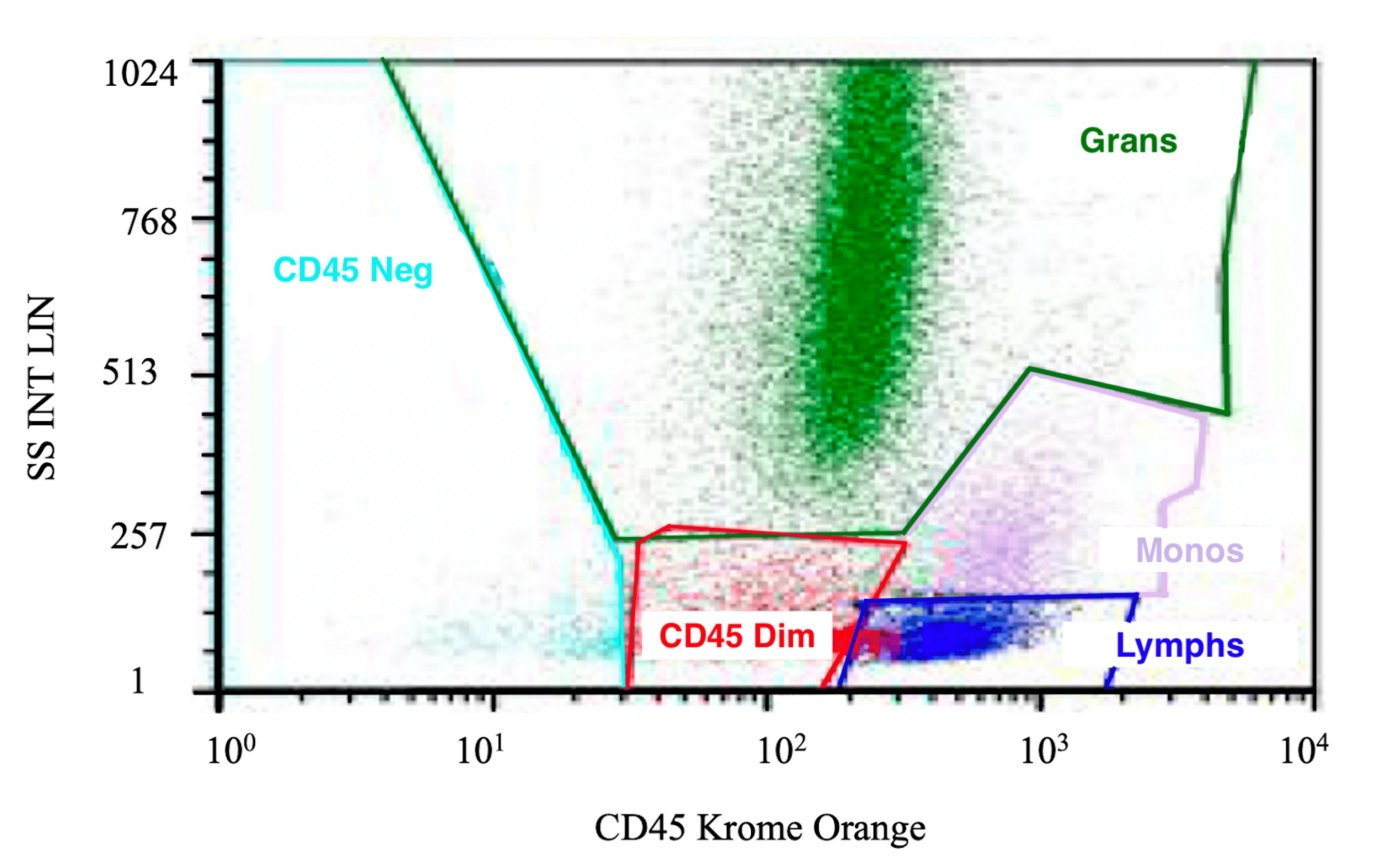
Flow cytometry demonstrating left-shifted myeloid maturation in dengue-associated cytopenia Representative flow cytometry dot plot showing a spectrum of hematopoietic populations. Granulocytes (Grans), monocytes (Monos), lymphocytes (Lymphs), and CD45-dim progenitor cells are identified. The presence of a left-shifted myeloid maturation pattern supports a reactive bone marrow process in the setting of acute dengue virus infection. No abnormal blast population is seen. CD: cluster of differentiation; SS INT LIN: side scatter intensity linear scale

Bone marrow analysis revealed a mildly hypocellular marrow (40%) with erythroid hypoplasia and megakaryocytic hyperplasia, without evidence of increased blasts or malignancy, as shown in Figure 1. Flow cytometry revealed a left-shifted myeloid maturation pattern, consistent with a reactive process (Figure 2). Cytogenetic analysis identified a Robertsonian translocation between chromosomes 13 and 14 (45, XX, der(13;14)(q10;q10)), a likely constitutional variant unrelated to the patient’s acute presentation. Fluorescence in situ hybridization (FISH) analysis was negative for common hematologic malignancy-associated chromosomal abnormalities. Collectively, these findings supported a secondary reactive marrow process, most consistent with infection-associated cytopenias in the setting of dengue virus infection.

By hospital day five, the patient’s platelet count, white blood cell count, and liver enzymes began to normalize. Hematemesis, hematuria, and fever resolved without the need for transfusion. She was discharged in stable condition with outpatient follow-up scheduled to review biopsy and serology results. The patient and her family were educated on signs of worsening illness and advised to follow up with her primary care provider for continued monitoring.

## Discussion

Dengue fever is a globally significant arboviral disease with an expanding geographic footprint, including the southern United States. Although most cases present with classic features, fever, myalgias, and rash, dengue can manifest atypically, particularly in secondary infections or endemic areas where host and viral factors interact in unpredictable ways [1,3]. Outbreak investigations along the Texas-Tamaulipas border have documented both dengue and dengue hemorrhagic fever, including the first reported locally acquired DHF case in Brownsville, Texas, in 2005, and a concurrent outbreak in northern Mexico in which nearly 18% of dengue cases met WHO criteria for DHF [4,14]. Diagnosis is typically presumptive in tropical regions, though definitive diagnosis requires immunodiagnostic or viral studies [15]. In this case, the patient's positive NS1 antigen test obtained in Mexico heightened clinical suspicion for dengue. NS1 testing has high specificity (>95%) but variable sensitivity depending on the day of illness and serotype, with optimal performance in the first week of infection [1]. Treatment is primarily symptomatic and supportive, focusing on rehydration for mild-to-moderate cases and intravenous fluids and blood transfusions for severe cases [15]. In some instances, dengue can progress to life-threatening conditions such as dengue hemorrhagic fever or dengue shock syndrome.

Our patient’s presentation was notable for hematemesis, hematuria, severe leukopenia, and neutropenia in the absence of hemoconcentration or plasma leakage. These findings, while uncommon, have been reported in dengue cases, particularly during the early viremic phase when immune-mediated suppression of hematopoiesis may be pronounced [12,13]. Early recognition of her hemorrhagic symptoms, despite the absence of severe thrombocytopenia, was critical in reinforcing proper care for potential bleeding risks regardless of her initial platelet count. Her thrombocytopenia developed during hospitalization, emphasizing the importance of daily physical examinations and ongoing communication with her family regarding the progression of dengue fever. In this case, the family expressed significant concern over her thrombocytopenia and requested multiple platelet infusions. Importantly, the patient improved without blood product transfusion, aligning with current evidence that routine platelet transfusions are not beneficial in dengue unless active bleeding or invasive procedures are involved [15]. The family was informed of this approach and reassured accordingly. Given the overlapping epidemiology of febrile illnesses in South Texas, other infectious etiologies, such as rickettsial disease, were also considered. The patient received empiric doxycycline; however, the absence of rash, lack of tick exposure history, and her improvement with supportive care alone made rickettsial disease unlikely and supported dengue as the final diagnosis.

Finally, consulting Heme/Onc due to the patient's down-trending thrombocytopenia, leukopenia, and neutropenia between days one to four was to ensure that there were no other causes of these cytopenias. The peripheral blood smear showed teardrop cells and large platelets. The peripheral smear findings in conjunction with the cytopenia suggested that the bone marrow may be unable to meet the demands of normal blood cell production, necessitating further investigation to determine the underlying cause [16]. The BM results demonstrated a left-shifted myeloid maturation pattern, suggesting a reactive or secondary process, such as an infectious or inflammatory disorder, autoimmune disease, or peripheral destruction/consumption. Taken together with the peripheral smear findings of teardrop cells and large platelets, these results were more consistent with marrow suppression from viral infection rather than peripheral destruction as the primary driver of cytopenias. Additionally, to rule out the possibility of primary marrow processes, a cytogenetic study and a next-generation sequencing (NGS) study were sequentially performed. Incidentally, the cytogenetic studies identified a Robertsonian translocation involving chromosomes 13 and 14 (45, XX, der(13;14)(q10;q10)), most likely representing a constitutional abnormality rather than an acquired change [17]. This finding is not associated with a specific hematologic disorder and did not put the patient at an increased risk for her current complications [17]. Overall, the results were consistent with the patient’s presentation of an infectious disorder (dengue fever) and added reassurance to the patient and her family that no primary marrow processes were causing her down-trending cytopenias.

Beyond the clinical implications, this case highlights a critical public health issue: dengue is increasingly endemic in the RGV, yet remains underrecognized [4,5]. Local clinicians must maintain a high index of suspicion for dengue in patients with recent travel to or residence in border regions. Moreover, the presence of severe hematological abnormalities without classic plasma leakage features should not preclude a dengue diagnosis, and timely supportive care should not be delayed while awaiting confirmatory testing [18].

## Conclusions

This case highlights an atypical presentation of dengue fever in a 22-year-old Hispanic female who developed severe leukopenia, neutropenia, and hematemesis in the absence of plasma leakage. Her recovery with supportive care alone underscores the importance of clinical vigilance, close monitoring, and adherence to evidence-based management protocols. In regions with emerging endemicity, such as South Texas, recognizing atypical dengue presentations is critical to guiding care and avoiding unnecessary interventions. This case also emphasizes that dengue can present with severe cytopenias and upper gastrointestinal bleeding, expanding the differential for hematemesis. Flow cytometry and bone marrow biopsy were essential in excluding hematologic malignancy, and this diagnostic approach should be considered in similar presentations. Ultimately, clinicians must maintain a high index of suspicion for dengue in patients returning from endemic areas to ensure timely and appropriate management. Simply put, this case reminds us that dengue does not always follow the textbook, and careful supportive care is usually enough to help patients recover.
